# Molecular mechanisms of synergistic action of Ramucirumab and Paclitaxel in Gastric Cancers cell lines

**DOI:** 10.1038/s41598-020-64195-x

**Published:** 2020-04-28

**Authors:** Maria Grazia Refolo, Claudio Lotesoriere, Ivan Roberto Lolli, Caterina Messa, Rosalba D’Alessandro

**Affiliations:** 1 Laboratory of Cellular and Molecular Biology, Department of Clinical Pathology, National Institute of Gastroenterology, “Saverio de Bellis” Research Hospital, Castellana Grotte (BARI), 70013 Italy; 2 Medical Oncology Unit, National Institute of Gastroenterology, “Saverio de Bellis” Research Hospital, Castellana Grotte (BARI), 70013 Italy

**Keywords:** Gastric cancer, Gastric cancer

## Abstract

Ramucirumab is approved both as monotherapy and in combination with Paclitaxel for advanced gastric cancer in patients with disease progression after chemotherapy. In tumor cells, the VEGFA-VEGFR2 binding activates autocrine survival and migration signaling in angiogenesis independent manner. The present *in vitro* study investigated the effects of single and combined treatments with Ramucirumab and Paclitaxel on cell growth and migration highlighting the mechanisms underlying the interaction between the two drugs in gastric cancer cells. Cell growth and motility were investigated in human gastric cancer cell lines characterized by different tumorigenicity. The inhibitory effect on cell growth exerted by both drugs was potentiated by their combination and was synergistic. Ramucirumab was able to enhance the inhibitory effect exerted by Paclitaxel on cell cycle progression. A synergistic action was also observed in the expression of proteins crucial for cell motility, microtubule organization and epithelial-mesenchymal transition. Furthermore, synergistic inhibition of VEGFR2 expression was obtained by the drug combination. These findings highlighted the importance of the combined treatment to strongly inhibit all the main molecules of both PI3K/Akt/mTOR and MAPK pathways thus preventing possible reactivations due to cross-talk phenomena. The combined treatment with Ramucirumab seems to be a promising option to overcome the Paclitaxel resistance.

## Introduction

VEGFR2 plays a crucial role in gastric cancer pathogenesis and progression and Ramucirumab (Ram), a monoclonal antibody against VEGFR2, is the first antiangiogenic agent with demonstrated activity against advanced gastric cancer. Based on the results obtained by two different phase III studies, Ram is approved both as monotherapy and in combination with Paclitaxel (PTX) for this malignancy in patients with disease in progression after a preceding therapy based on platinum and fluoropyrimidin^1–5^.

The proangiogenic actions of VEGFs in endothelial cells are mediated primarily through the binding and activation of VEGFR2^6^ and retrospective studies considered VEGF and its receptors as possible biomarkers in gastric carcinoma^7–9^. Moreover, the recent discovery of the production of different VEGF ligands and of the expression of VEGFR1, VEGFR2 and VEGFR3 in epithelial cancer cells, suggests a direct role for these ligands and their receptors in the autocrine control of some biological processes. In epithelial tumor cells, the binding of VEGFA, the main ligand of VEGFR2, activates downstream survival and migration signaling pathways represented by PI3K/Akt/mTOR and MAPK cascades in a cell autonomous and angiogenesis independent manner^10–13^.

The combined treatment with Ram seems to be a promising option to prevent the resistance to PTX also in recurrent and metastatic gastric cancer patients receiving taxane-based first-line palliative chemotherapy^14^. PTX is known to interfere with microtubule architecture by binding to β-tubulin, thereby blocking cell cycle progression at the G2/M phase and inducing apoptosis. PTX exerts its action also by promoting tubulin polymerization and inhibiting tubulin depolymerization. Microtubule disorganization, decreased responses to antimitotic treatments, and apoptotic process failure can induce acquired resistance to PTX in advanced gastric cancer^15–19^.

The present study attempted to investigate the biological processes and the molecular mechanisms that underlie the interaction between the two drugs in human gastric cancer cell lines. The analysis was focused on the evaluation of single and combined effects of Ram and chemotherapy agent on cell proliferation, apoptosis and migration. The expression of VEGFA and VEGFR2 was investigated at basal level and after drug treatments. Moreover, the analysis was extended to some of the main factors of the PI3K/Akt/mTOR and MAPK pathways as well as proteins regulated by these signaling pathways involved in the regulation of cell cycle and cell motility.

## Results

### Differential expression of VEGFA and VEGFR2 in gastric cancer cell lines

**Figure 1:**
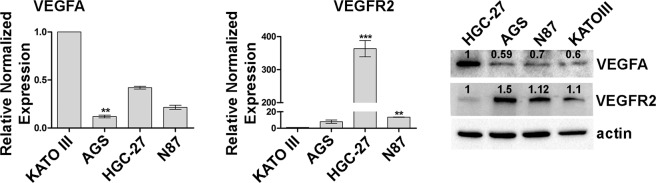
Differential expression of VEGFA and VEGFR2 in gastric cancer cell lines. mRNA and protein expression of VEGFA and VEGFR2 were evaluated in KATO III, AGS, HGC-27 and NCI-N87 [N87] human gastric cancer cell lines using Real Time-PCR and Western Blotting analysis respectively. The data of three independent Real Time-PCR experiments were plotted in the graphs and expressed as mean ± SD. **p < 0.001; ***p < 0.0001. A representative Western Blotting image showing the expression levels of VEGFA and VEGFR2 is presented.

Four different human gastric cancer cell lines (HGC-27, NCI-N87 [N87], KATO III and AGS) were characterized by the expression of VEGFA and its receptor (VEGFR2) both at mRNA and protein level. Although the mRNA analysis detected he greatest content of VEGFA in KATO III cells, these data were not confirmed by the Western Blotting experiments that identified the highest VEGFA expression in HGC-27 cell line. Similarly, for VEGFR2 expression the highest amount of mRNA was revealed in HGC-27 cells while the highest expression at protein level was detected in AGS and N87 cells (Fig. 1).

### Ramucirumab potentiates the growth inhibition of Paclitaxel

**Figure 2:**
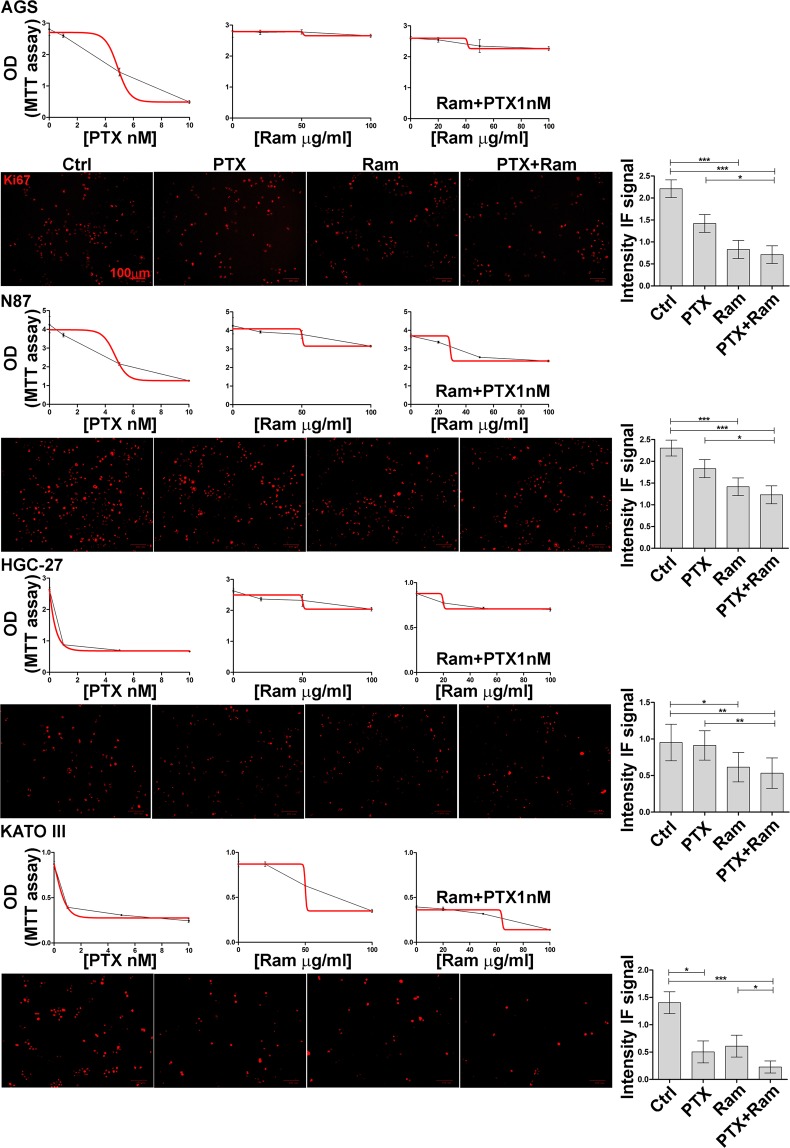
Dose response curves relative to each single or combined treatment and Ki-67 staining in gastric cancer cell lines. AGS, NCI-N87 [N87], HGC-27 and KATO III human gastric cancer cell lines were cultured in medium containing different concentrations of PTX (1, 5, 10 nM) and Ram (20, 50, 100 μg/ml) used alone or in combination for 48 h. Dose-response curves for each drug or drug combination were generated. The results of three independent experiments were expressed as mean ± SD. Representative pictures of Ki-67 staining were shown (Scale bar: 100 µm), graphs on the right represent the average value of IF intensity signals obtained from three independent experiments. *p < 0.05; **p < 0.001; ***p < 0.0001.

All the cell lines investigated were cultured in presence of Ram and/or PTX. A range of concentrations (as described in Materials and Methods section) was examined for each of the two drugs in cell proliferation assay evaluated after 48 h by MTT. Dose response curves relative to each single or combination treatment were performed and the MTT optical density values were plotted in the graphs shown in Fig. 2.

Addition of increasing Ram concentrations caused a strong effect on cell growth inhibition in all the combinations tested. Data showed that 100 µg/ml Ram enhanced the inhibition exerted by 1 nM PTX lowering its half maximal inhibitory concentration (IC50) in all cell lines investigated with different intensity (5 nM vs 5.8 nM in AGS, 5.7 nM vs 6.4 nM in N87, 2.9 nM vs 3.1 nM in HGC-27 and 3.9 nM vs 5.4 nM in KATO III) (Table 1).

**Table 1:** IC_50_ values relative to each single or combined treatment.

	AGS IC_50_	N87 IC_50_	HGC-27 IC_50_	KATO III IC_50_
PTX (single drug treatment)	5.8	6.4	3.1	5.4
Ram (single drug treatment)	1030	200	235	86
PTX (in combination with Ram 100 µg/ml)	5	5.7	2.9	3.9

Each value derived from the linear curve equation of the dose-response curve: y = mx + c (50= slope*IC_50_ + intercept). PTX concentration was expressed as nM and Ram as µg/ml.

The indicated concentrations were the lowest showing a significant effect in a single drug treatment and they have been subsequently used in all assays. According to Chou e Talalay method^20,21^, the Combination Indexes (CI) obtained for these drug combinations were ≤1, demonstrating that drugs exerted their effect synergistically (Table 2).

**Table 2:** Combination Index values relative to each combined treatment. Each value derived from Chou e Talalay method implemented in CompuSyn software.

AGS			N87		
PTX (nM)	Ram (µg/ml)	CI	PTX (nM)	Ram (µg/ml)	CI
1	20	0,86	1	20	0,97
1	50	0,73	1	50	0,46
1	100	0,68	1	100	0,4
5	20	1,48	5	20	1,15
5	50	1,27	5	50	0,89
5	100	1,06	5	100	0,74
10	20	0,5	10	20	0,84
10	50	0,6	10	50	0,6
10	100	0,45	10	100	0,56
**HGC-27**			**KATO III**		
PTX (nM)	Ram (µg/ml)	CI	PTX (nM)	Ram (µg/ml)	CI
1	20	0,53	1	20	0,91
1	50	0,28	1	50	0,43
1	100	0,25	1	100	0,39
5	20	0,39	5	20	1.05
5	50	0,23	5	50	0,84
5	100	0,15	5	100	0,66
10	20	1,81	10	20	0.84
10	50	0,46	10	50	0,6
10	100	0,53	10	100	0,5

**Figure 3:**
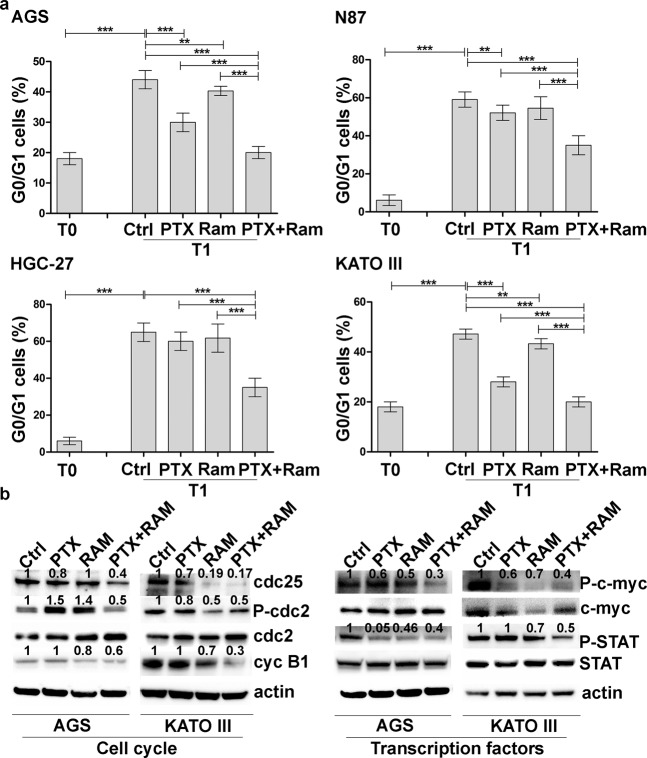
Effects of Ramucirumab and/or Paclitaxel on cell cycle progression in gastric cancer cell lines. (**a**) AGS, NCI-N87 [N87], HGC-27 and KATO III human gastric cancer cells were synchronized in the G2/M phase of the cell cycle (T0). 1 nM Paclitaxel and 100 μg/ml Ram alone or in combination were administrated to a subgroup of cells processed after 3 h from block release (T1). The percentage of cells in the subsequent G0/G1 phase was evaluated and plotted in the graphs. The results of three independent experiments, expressed as mean ± SD, were plotted in the relative graphs. *p < 0.05; **p < 0.001; ***p < 0.0001. (**b**) The expression of activated cdc25A, cdc2, Cyclin B1, c-myc and STAT3 was analyzed in AGS and KATO III cells after 48 h of Ram and/or PTX treatment. Three independent experiments were performed and a representative Western Blotting image is shown.

To determine the growth rate of treated cell population, the effects of single or combined treatments were investigated also by Ki67 staining. The mean values relative to the intensity of fluorescent signal were plotted in the graph for each cell line and showed in Fig. 2. The results obtained for HGC-27, N87 and AGS cells revealed that the inhibitory action of Ram was more pronounced respect to that of PTX and this inhibition was even more marked after the combined treatment. In KATO III cells PTX exerted a higher inhibitory effect respect to Ram. It is interesting to note that in this cell line the combined treatment was the most effective leading to a substantial decrease in the fluorescence signal. The effect caused by Ram on growth inhibition mediated by PTX was also evaluated on cell cycle progression. In AGS and KATO III cells, PTX was able to reduce the progression from the G2/M phase of cell cycle to the subsequent G0/G1 despite the low concentration used after 3 h (T1) from block release (T0). Although Ram was ineffective in inhibiting progression, its effect was enhanced when administrated in combination with PTX. In order to investigate the molecular mechanisms underlying cell cycle progression, some of the main factors involved in the activation of the mitosis-promoting factor (MPF) complex, crucial for the G2/M progression, were evaluated (Fig. 3a).

Western Blotting analysis performed in AGS and KATO III cells revealed a huge decrease in the expression of activated cdc25A, P-cdc2 and Cyclin B1 after 48 h of Ram/PTX combined treatment. Moreover, the activation status of two transcription factors, c-myc and STAT3, implicated at different levels in determining the proliferation rate, was investigated. Both phosphorylated factors showed a decrease in expression levels after single drug administration and more markedly in combined treatment (Fig. 3b).

**Figure 4:**
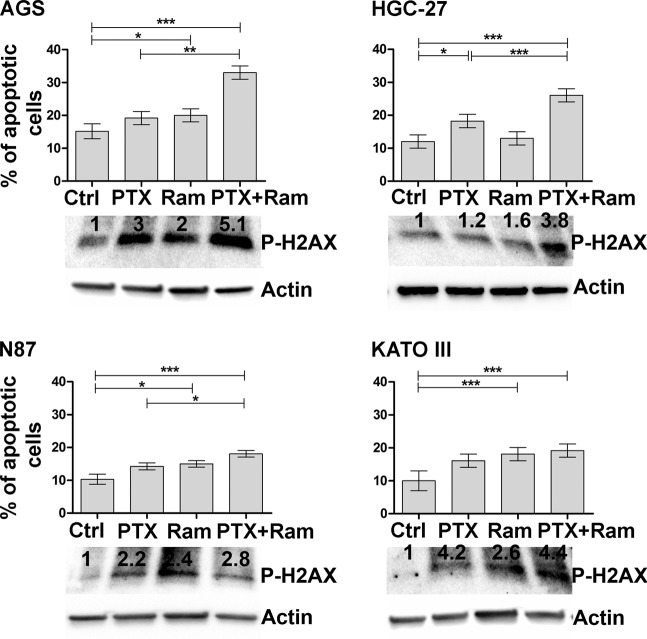
Effects of Ramucirumab and/or Paclitaxel on cell apoptosis related to DNA damage in gastric cancer cell lines. Muse Annexin V Cell Assay and Phospho-Histone H2AX (Ser139) Western Blotting for AGS, NCI-N87 [N87], HGC-27 and KATO III human gastric cancer cell lines assessed after 48 h of 100 µg/ml Ram and/or 1 nM PTX treatment. Apoptosis results deriving from three independent experiments were expressed as means ± SD and reported in the graphs. The expression of Phospho-Histone H2AX was analyzed by Western Blotting and a representative image is shown. *p < 0.05; **p < 0.001; ***p < 0.0001.

Next, the effects of Ram on PTX-mediated apoptosis after 48 h were investigated. The same drug concentrations as for the proliferation experiments were used to evaluate the Annexin V. Both drugs caused a modest effect on the induction of the apoptotic process, however the combined treatment enhanced the stimulatory effect. Averagely, the percentage of apoptotic cells was doubled respect to untreated control cells in all investigated gastric cancer cell lines (Fig. 4).

Moreover, DNA damage was assessed in same experimental conditions by analyzing the expression levels of P-H2AX protein. Western Blotting analysis revealed an increase in the protein phosphorylation levels after single treatment in all cell lines, this effect was even more evident after combined treatment especially in AGS and KATO III cells.

### Ramucirumab potentiates the inhibitory effects of Paclitaxel on cell migration and actin polymerization

**Figure 5:**
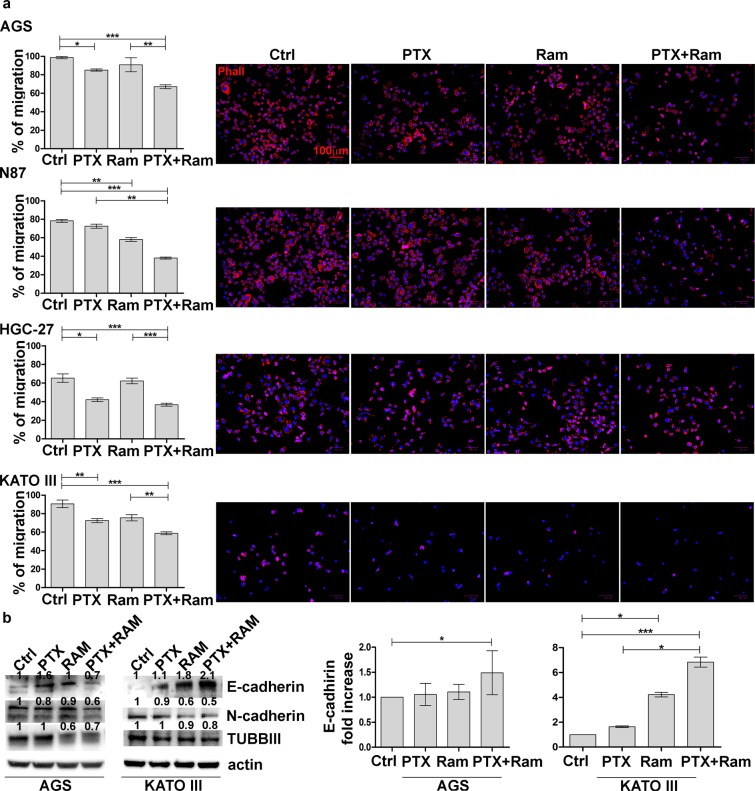
Effects of Ramucirumab and/or Paclitaxel on cell migration and actin polymerization in gastric cancer cell lines. AGS, NCI-N87 [N87], HGC-27 and KATO III human gastric cancer cell lines were cultured for 48 h with 100 µg/ml Ram and/or 1 nM PTX. (**a**) The results obtained by migration assay performed on fibronectin coated wells is shown on the left. The percentage of migration were calculated at the time T0 and after 48 h (T2). The 100% represented the detection zone completely closed. The experiments were performed in triplicate and the mean values ± SD were plotted in the relative graph. *p < 0.05; **p < 0.001; ***p < 0.0001. The representative images of cell staining with DyLight 554 Phalloidin are presented on the right. Scale bar: 100 µm. **(b**) The expression of β-tubulin III (TUBB III) and EMT protein E-Cadherin and N-Cadherin after 48 h of Ram and/or PTX treatment was analyzed by Western Blotting in three independent experiments and a representative image is shown on the left. On the right are presented the data of three independent Real Time-PCR experiments of E-Cadherin plotted in the graphs and expressed as mean ± SD. *p < 0.05; ***p < 0.0001.

To test the effects of Ram on PTX-mediated inhibition of cell migration, cells treated as previously indicated were cultured onto Oris plates. Microscopic analysis of cell migration on fibronectin matrix was performed after the stoppers (T0) removal and at subsequent time intervals. In the graphs were reported migration data after 48 h (T2). The treatment with Ram or PTX exerted a stronger inhibitory effect in cell lines characterized by low motility such as HGC-27 and N87 compared to those characterized by greater motility. However, an average reduction of 50% of the migration rate was observed in the cells treated with the combination of the two drugs compared to the control in all cell lines investigated (Fig. 5a). Moreover, it was examined whether the combined treatment also had efficacy on the cytoskeleton organization. DyLight 554 Phalloidin staining revealed that both Ram and PTX, administrated for 24 h alone or in combination, caused significant reduction and depolymerization of actin in the cells. As a consequence, a loss of F-actin fibers in the cytoplasm was evident (Fig. 5a). The action of the two drugs in the modulation of the cytoskeletal fiber organization was evidenced also by the Western Blotting analysis of β-tubulin III expression. This protein was particularly inhibited by the combined treatment although Ram itself was also able to exert an evident inhibitory effect (Fig. 5b). Epithelial–Mesenchymal Transition (EMT) protein expression analysis revealed that epithelial marker E-cadherin resulted overexpressed after single drug treatment in both cell lines and major effects were obtained after combination of PTX and Ram in KATO III cells. This result was confirmed at mRNA level. On the other hand, the mesenchymal marker N-cadherin resulted down regulated mainly after combined drug treatment in both cell lines (Fig. 5b).

### Ramucirumab and Paclitaxel modulate the cell expression of VEGFA and VEGFR2 and the VEGFA medium levels

**Figure 6:**
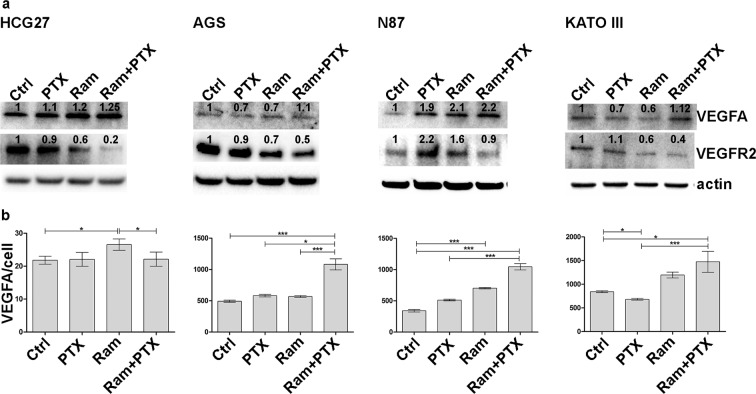
Ramucirumab and Paclitaxel modulate the cell expression of VEGFA and VEGFR2 and the VEGFA medium levels. (**a**) A representative Western Blotting image of VEGFA and VEGFR2 expression is presented. the analysis was performed after 48 h of 100 µg/ml Ram and/or 1 nM PTX treatments in HGC-27, AGS, NCI-N87 [N87] and KATO III cells. (**b**) VEGFA levels in the cell culture medium were measured 48 h after each drug treatment and the respective values divided by the number of viable cells are reported in the graphs. The results of three independent experiments were expressed as mean values ± SD. *p < 0.05; ***p < 0.0001.

To explore the mechanism underlying the modulatory effects of Ram and PTX on VEGFA and VEGFR2 expression, the protein levels of their expression were investigated after 48 h of single or combined treatments. The VEGFA expression levels were unchanged in AGS and KATO III cells, while an increase in its expression was observed in HGC-27 and N87 both after single or dual drug treatments. By contrast, the analysis of VEGFR2 expression revealed a significant decrease in its expression after Ram and especially after combined treatment in all cell lines (Fig. 6a).

The free VEGFA amount was measured in cell culture medium in the same experimental condition, by enzyme-linked immunosorbent assay. The VEGFA amount referred to single cell was increased respect to control untreated cells after Ram treatment in all analyzed cell lines. This effect was enhanced by combined treatments in AGS, N87 and KATO III cells (Fig. 6b).

### Ramucirumab and Paclitaxel modulate synergistically MAPK and PI3K/Akt signaling inhibition

**Figure 7:**
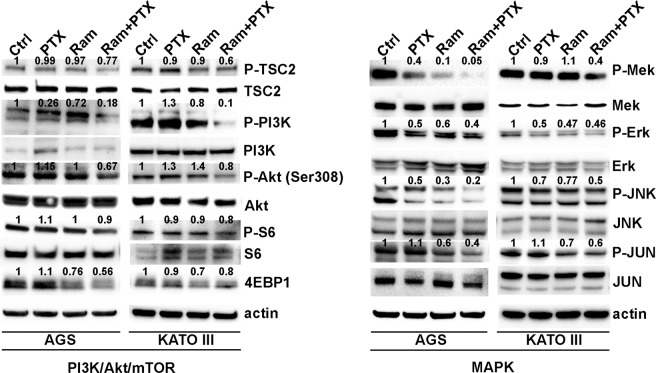
Effects of Ramucirumab and/or Paclitaxel on MAPK and PI3K/Akt signaling in gastric cancer cell lines. The expression of main molecules at the cross-road of MAPK and PI3K/Akt/mTOR pathways was analyzed by Western Blotting after 48 h of 100 µg/ml Ram and/or 1 nM Paclitaxel treatments in AGS and KATO III cells. Three independent experiments were performed and a representative Western Blotting result is shown.

The modifications of the main molecules at the cross-road of MAPK and PI3K/Akt/mTOR pathways, both activated by the VEGFA/VEGFR2 binding, were investigated. Western Blotting analysis was performed in AGS and KATO III cells treated with PTX and Ram alone or in combination for 48 h. The phosphorylation levels of TSC2, PI3K, AKT, S6 and 4EBP1 were evaluated in both cell lines. The results indicated that PTX was almost ineffective in modulating the phosphorylation levels of all analyzed proteins of PI3K/Akt/mTOR signaling pathway, while Ram exerted a weak inhibitory effect that was strongly potentiated by the combination with PTX in both cell lines. The significant inhibitory effect of PTX on the modulation of the proteins involved in MAPK signaling pathways such as MEK, Erk, JNK and JUN was detected in both cell lines. Decrease in the phosphorylation levels of these proteins was notable also in the presence of Ram and the effect was enhanced when the both drugs were contemporarily administrated (Fig. 7).

Erk phosphorylation was evaluated relative to the total Erk expression by Muse MAPK Activation Dual Detection Kit in AGS, N87, HGC-27 and KATO III cell lines. Addition of Ram to the cultures treated with PTX caused a greater decrease in P-ERK levels, compared to single drug treatments in all cell lines investigated (Fig. S1) These results were in accordance with previous Western Blotting data obtained for AGS and KATO III cells (Fig. 7). Similarly, the activation of PI3K/Akt pathway in the same experimental conditions was investigated by Muse PI3K Activation dual detection Kit. Data revealed that neither Ram or PTX had significant effect on the percentage of P-Akt (Ser473) activation, but a decrease in P-Akt levels respect to untreated cells was detected when these two drugs were added in combination (Fig. S1).

## Discussion

Among the metastatic gastric cancer patients that were progressed after a first-line therapy, those with better performance status and acceptable organ function could be considered candidates for a second-line therapy^1–3^. Ram is approved both as monotherapy and in combination with PTX for advanced gastric cancer patients with disease in progression after platinum and fluoropyrimidin therapy^4,5^. Furthermore, a network meta-analysis compared the effectiveness of the second-line Ram plus PTX co-treatment with Best Supportive Care (BSC) and docetaxel treatment respectively. In both cases significant improvement of PFS derived from Ram/PTX combined treatment^22^. Unfortunately, Ram in combination with fluoropyrimidine and cisplatin has not been proven to be effective as chemotherapeutic agents administrated in front line therapy as documented by RAINFALL study^23^. Nevertheless, the early administration of an active regimen such as the combination of Ram and PTX, after the first-line therapy and before disease progression might prolong the benefit of first-line treatment with an improvement of the quality of life. This hypothesis is actually tested in the ongoing ARMANI study^24^. Finally, interesting data from KEYNOTE-098 phase I study was reported. Actually, the study showed that Ram in combination with an immunotherapeutic agent such as the anti-PD1 Pembrolizumab, applied as second or later line therapy, had a controllable safety profile with antitumor activity in patients previously treated for advanced GC/GEJC, non-small-cell lung cancer and urothelial carcinoma^25^.

The combined regimen represents a promising option to increase the efficacy of PTX and to prevent its resistance also in patients receiving taxane-based first-line palliative chemotherapy^14^. Although PTX is widely used as a first-line agent in chemotherapies, several studies indicate factors such as microtubule organization, EMT phenotype and apoptotic resistance as the main causes of resistance to treatment with this drug^15–18,26–30^. In epithelial tumor cells, the binding of VEGFA to VEGFR2 sustains autocrine loop affecting cell growth and migration. Ram binds the receptor about eight times more effectively than VEGFA^31^, thus targeting not only the neo-angiogenesis in endothelial cells^6^ but also the growth and motility of tumor cells^10–13^. Although the effects of Ram as an anti-angiogenic drug are now well documented, little is known about its role in inhibiting the growth and motility of gastric cancer cells both as single agent and in combination with chemotherapeutic drugs such as PTX. The present study gives significant evidence of the synergistic effect between Ram and PTX drugs. In particular, the effects of single and combined treatments on cell growth and migration as well as the molecular mechanisms that underlie the interaction between the two drugs were investigated in four gastric cancer cell lines characterized by specific genetic alterations linked with pro-tumoral processes. Specifically, the amplification of HER2 oncogene, closely related to the activation of PI3K/Akt or MAPK pathway, is found in N87 cell line deriving from intestinal form of GC^32^. On the contrary, inactivation of E-Cadherin/b-catenin system, amplification of K-sam and c-met oncogenes are mostly characteristics of KATO III and AGS cells that represent the diffuse type of GC^33^. HGC-27 cells are instead defined as PTEN mutant GC cells^34^. These genetic hallmarkers make AGS and KATO III cell lines good *in vitro* models for the evaluation of the effects of the two drugs on cell growth and motility, on the reversal of the EMT and on the main factors involved in PI3K/Akt/mTOR and MAPK pathways that lead to tumor growth and progression. Therefore, these cell lines represent a valid approach for the biologic and pharmacological study of the heterogeneous human GC.

In the present study, the GC cell lines were characterized by the expression level of VEGFA and its receptor (VEGFR2). The highest VEGFA and the lowest VEGFR2 protein levels were present in HCG-27 cells, while AGS cells were characterized by the highest VEGFR2 levels. Dose response results showed that, regardless of the expression levels of VEGFR2, the inhibitory effect on cell growth exerted by both drugs was potentiated by their combination and was clearly synergistic (CI ≤ 1)^20,21^. The Ki-67 staining confirmed the anti-proliferative effects achieved by co-treatment with both drugs. Comparing to PTX, Ram showed a greater inhibiting capacity on cell proliferation and was able to significantly enhance the anti-proliferative effect of PTX especially in AGS and KATO III cells. The study of cell cycle progression revealed that although Ram itself was ineffective in inhibiting the progression from the G2/M phase to the subsequent G0/G1phase of cell cycle, it was able to enhance the expected inhibitory effects of PTX on cell cycle progression in all cell lines investigated. However, the effect again was more pronounced in AGS and KATO III cell lines. For this reason, the expression analysis of some of the main factors involved in the activation of the MPF^35^ complex was restricted to these two cell lines. The MPF was a complex crucial for the G2/M progression, the results revealed a huge decrease in the expression of activated cdc25A, cdc2 and Cyclin B1 after Ram/PTX combined treatment^18^. The increase of P-H2AX levels after single and combined treatments in all cell lines, demostrated by Western Blotting, supported the idea that induction of apoptosis and cell cycle arrest are possible outcome of DNA damage. Moreover, despite the modest effects caused by single-drug treatments, a reduction of 50% of the migration rate was observed in the cells treated with drugs combination in all cell lines investigated. DyLight 554 Phalloidin staining revealed that both Ram and PTX, administrated alone or in combination, caused a significant reduction and depolymerization of F-actin in the cells. The synergistic effects were evidenced also by the analysis of β-tubulin III protein whose expression was significantly inhibited upon dual drug treatment. EMT protein expression analysis revealed that while epithelial marker E-Cadherin was overexpressed, the mesenchymal marker N-Cadherin was down regulated after combined drug treatment^18,26^. The VEGFA expression levels were unchanged in AGS and KATO III cells while an increase was observed in HGC-27 and N87 after single or dual drug treatments. On the other hand, Ram exerted its inhibitory effect also by reducing the VEGFR2 expression and also in this case the simultaneous administration of the two drugs led to further decrease in VEGFR2 expression level. This effect was particularly relevant considering the pivotal role of the receptor in regulating the described autocrine mechanism. The treatment with Ram caused an increase of free VEGFA amount in cell supernatant. This accumulation of the ligand in the medium is expected result considering that VEGFR2 receptor binding sites are occupied by Ram^36^. Surprisingly, a further increase of free VEGFA was observed after dual drug treatment which is probably associated with the decrease of the VEGFR2 binding sites, that occurred in these experimental conditions. To get an insight into downstream molecular modifications caused by Ram/PTX interaction the main molecules at the cross-road of PI3K/Akt/mTOR^37^ and MAPK^11^ pathways, both activated by the VEGFA/VEGFR2 binding, were analyzed. Regarding the PI3K/Akt/mTOR signaling, the results indicated that low concentrations of Ram had a weak inhibitory effect on the signal pathway activity that was strongly potentiated by the combination with PTX, which by itself was almost ineffective in single treatments. Phosphorylation level of PI3K/Akt/mTOR pathway-related proteins was used as indicator for the signaling pathway activity. The most significant decrease in activation levels was achieved for P-TSC2 and P-PI3K as a result of the combined treatment. The inhibitory effects of Ram and PTX were significant in modulation of the proteins involved in MAPK signaling and the synergism of the two drugs resulted in a further decrease in phosphorylation levels, especially of P-Mek and P-JNK proteins.

Considering the key role of angiogenesis in the resistance mechanisms of several drugs including the new immunotherapeutic agents and chemotherapy, the studies focusing on the molecular mechanisms underlying drug interaction are of particular interest. The combination of Ram and PTX represents the standard second line therapy for patients with metastatic GC. Overall, the presented data underlined the importance of this combined treatment in order to strongly inhibit all the main molecules of both signal pathways thus preventing possible reactivations due to cross-talk phenomena. The present study has clearly revealed the synergy behind the interaction between the two drugs in cellular processes that are crucial for tumor growth and progression. However, further research will be necessary to evaluate possible differences between these drugs and other inhibitors of the VEGF pathway or taxanes other than PTX.

## Materials and Methods

### Cells and drugs

AGS, KATO III, NCI-N87 [N87] human gastric cancer cell lines were purchased and authenticated by American Type Culture Collection (Manassas, Virginia, USA) and HGC-27 by Interlab Cell Line Collection (Genova, Italy). All cell culture components were purchased from Sigma-Aldrich (Milan, Italy). PTX was purchased from Teva Italia S.r.l. (Milan, Italy) and Ram from Eli Lilly (Utrecht, Nederland).

### Cell culture

Gastric cancer cell lines were cultured in Dulbecco’s Modified Eagle Medium (DMEM) supplemented with 10% fetal bovine serum (FBS), 100 U/ml penicillin and 100 µg/ml streptomycin. The cells were incubated at 37 °C in a humidified atmosphere containing 5% CO_2_ in air^38^.

### Gene expression analysis

Total RNA was extracted from AGS, KATO III, NCI-N87 [N87] and HGC-27 cells using the Qiagen RNeasy Mini Kit (Qiagen, Hilden, Germany) according to the manufacturer’s instructions. Samples were retro-transcribed using the iScript Advanced cDNA Synthesis Kit (Bio-Rad Laboratories, California, USA). The SsoAdvanced PreAmp Supermix and PrimePCR PreAmp for SYBR Green Assay (Bio-Rad Laboratories, California, USA) were used as indicated in the users’ guide to provide unbiased preamplification reactions regardless of VEGFR2, enabling more copies of genes to be obtained from a limited source. Real Time-PCRs for the evaluation of VEGFA, VEGFR2 and E-Cadherin expression were carried out in triplicate using the SsoAdvanced Universal SYBR Green Supermix (Bio-Rad Laboratories, California, USA) on a CFX96 Touch Real-Time PCR Detection System (Bio-Rad Laboratories, California, USA) according to the manufacturer’s instructions. Pre‐validated PrimePCR Template for SYBR Green Assay (Bio-Rad Laboratories, California, USA) were used for reactions. Relative quantification was done using the ddCT method.

### Western Blotting

Western Blotting analysis was performed as previously described^38^ in AGS and KATO III cells. Primary antibodies were directed against the following proteins: VEGFA and beta III Tubulin (abcam, Cambridge, UK), VEGF Receptor 2, Phospho-Tuberin/TSC2 (Ser939), Tuberin/TSC2, Phospho-PI3 Kinase p85 (Tyr458)/p55 (Tyr199), PI3 Kinase p110α, Phospho-Akt (Thr308), Akt (pan), Phospho-S6 Ribosomal Protein (Ser235/236), S6 Ribosomal Protein, 4E-BP1, Phospho-MEK1/2 (Ser217/221), MEK1/2, Phospho-p44/42 MAPK (Erk1/2) (Thr202/Tyr204), p44/42 MAPK (Erk1/2), Phospho-SAPK/JNK (Thr183/Tyr185), JNK2, Phospho-c-Jun (Ser63), c-Jun, E-Cadherin, N-Cadherin, cdc25A, Phospho-cdc2 (Thr161), cdc2, Cyclin B1, Phospho-c-Myc (Ser62), c-Myc, Phospho-Stat3 (Ser727), Stat3, Phospho-Histone H2AX (Ser139) and β-actin (Cell Signaling, Beverly, MA, USA). The protein expression was quantified using the ImageJ software (http://rsb.info.nih.gov/ij/).

### Cell proliferation and drug synergy evaluation

AGS, KATO III, NCI-N87 [N87] and HGC-27 cells were treated with different concentrations of PTX (1, 5, 10 nM) and Ram (20, 50, 100 μg/ml) alone or in combination as indicated above. The concentration ranges were chosen using data reported in literature^18,39,40^.

After 48 h of treatment, the vital and proliferating cells ware estimated by colorimetric MTT 3-(4,5-dimethylthiazol-2-yl)-2,5-diphenyltetrazolium bromide test. Dose-response curves were calculated for each drug or drug combination and relative IC50 values were computed using Microsoft Office Excel. Each value was calculated from equation y = mx + c derived from the dose-response curve (with y = 50, the equation became: 50 = slope*IC50 + intercept)^41^.

Data derived from MTT assay were implemented in CompuSyn software (Biosoft, UK) based on the method defined by Chou and Talalay^20,21^. The Chou and Talalay approach considers drug effectiveness and the correlation dose-response for each drug. Results are reported as CI < 1, CI = 1 and CI > 1 and are referred to synergistic, additive and antagonistic effect, respectively. The lowest concentration of each drug with CI < 1 was used in all the subsequent experimental conditions, in particular cells were cultured with 1 nM PTX and 100 μg/ml Ram administrated singularly or in combination.

### Ki-67 immunofluorescence staining

Gastric cancer cells were cultured for 24 h in 1% FBS medium and then trypsinized, resuspended in 1% FBS medium and seeded in 96 multi-well plates. After attachment of the cells to the dishes, treatments were performed as previous described. The proliferating cells were evaluated by the staining with Ki-67. Immunofluorescence experiments were performed as previously described immunolabeling the cells with anti-human Ki-67 antibody (BioLegend INC. San Diego, CA) diluted 1:200 in Phosphate Buffered Saline (PBS) for 2 h in a humidified dark chamber. The analysis of the intensity of fluorescent signals was performed using the ImageJ software and the values were plotted in the relative graphs^41^.

### Cell cycle

AGS, KATO III, NCI-N87 [N87] and HGC-27 cells were synchronized by using 20 µg/ml nocodazole added to the medium. After 18 h of incubation, the medium containing nocodazole was replaced from fresh medium and cells were separated into two groups: one group was collected for cell cycle analysis (T0) and the second one continued culturing for 3 more hours as previous described. After 3 h (T1) the cells were processed by Muse Cell Cycle Kit (Millipore, Darmstadt, Germany) according to Muse Cell Analyzer protocol that determined the population of cells in the G0/G1, S and G2/M phases^41^.

### Apoptosis assay

Gastric cancer cells were cultured as described in “Cell proliferation and drug synergy evaluation” paragraph. The fluorescent signal emitted by dye conjugated antibodies was detected by Flow cytometry technology (Muse Cell Analyzer, Millipore, Darmstadt, Germany). The Muse Annexin V/Dead Cell Assay Kit (Millipore, Darmstadt, Germany) for quantitative analysis of live, early/ late apoptotic and dead cells was used with a Muse Cell Analyzer. Briefly, the assay utilizes Annexin V to detect phosphatidyl serine on the external membrane of apoptotic cells. 7-Amino-Actinomycin D (7-AAD) dead cell marker is also used^41^. The cells were then analyzed as described in the user’s guide.

### Migration assay

The migration assay was performed by Oris Cell Migration Assay (Platypus Technologies, Madison USA). Briefly, AGS, KATO III, NCI-N87 [N87] and HGC-27 cells were seeded onto Oris plates and after adhesion the stoppers that identify the detection zone were removed and cells were subjected to different drug treatments as described above. When the stoppers were removed the cells started to migrate into the detection zone. Photographs were taken of each well immediately after the stoppers removal (T0) and after 24 h (T1 not shown), 48 h (T2) and 72 h (T3 not shown). The values were expressed as percentage of migration, with 100% being when the detection zone was completely closed^41^.

### Phalloidin immunofluorescence staining

AGS, KATO III, NCI-N87 [N87] and HGC-27 cells were cultured for 24 h in 1% FBS medium and then were trypsinized, resuspended in 1% FBS medium and seeded in 96 multi-well plates. Immunofluorescence experiments were performed as previously described immunolabeling the cells in humidified dark chamber for 2 h with DyLight 554 Phalloidin (Cell Signaling, Beverly, MA, USA). Nuclei were stained with DAPI for 10 min in the dark. After rinsing with PBS, images were acquired with ZOE fluorescent cell imager (Bio-Rad, Milan, Italy)^42^.

### Measurement of VEGFA in cell culture medium

High sensitive Enzyme-Linked Immunosorbent Assay kit (Cloud-Clone Corp., Katy, TX USA) was used for *in vitro* quantitative measurement of human VEGFA in culture medium of HGC-27, AGS, NCI-N87 [N87] and KATO III cells treated as previously described, according to the instruction manual.

### MAPK activation assay

Muse MAPK Activation Dual Detection Kit (Millipore, Darmstadt, Germany), was used as previously described to measure simultaneously the levels of both the total and phosphorylated Erk in gastric cancer cell lines cultured for 15 min according to the above indicated treatments^41^.

### PI3K activation assay

Muse PI3K Activation dual detection Kit was used as previously described to detect simultaneously the levels of both the total and phosphorylated Akt in the four gastric cancer cell lines cultured in presence of the indicated treatments for 24 h^41^.

### Statistical analysis

Mann–Whitney non parametric test implemented in GraphPad Prism 5.0 software (La Jolla, CA, USA) was employed to evaluate the differences between two unmatched groups. A P value < 0.05 was considered statistically significant. All experiments were performed in triplicate and repeated three times. Data were shown as mean ± standard deviation (SD) in relative graphs.

## Supplementary information

Supplementary Figure S1.

## Data Availability

No datasets were generated or analysed during the current study.
