# Supplementary material for: Molecular mechanisms of synergistic action of Ramucirumab and Paclitaxel in Gastric Cancers cell lines
Source: Sci Rep. 2020 Apr 28;10:7162. doi: 10.1038/s41598-020-64195-x (PMC7188894; doi:10.1038/s41598-020-64195-x)
Supplement: Supplementary file 1 — Supplementary Figure S1. [file 41598_2020_64195_MOESM1_ESM.pdf]

Molecular mechanisms of synergistic action of Ramucirumab and Paclitaxel in Gastric Cancers cell lines

Maria Grazia Refolo[1], Claudio Lotesoriere[2], Ivan Roberto Lolli[2], Caterina Messa[1*] and Rosalba D'Alessandro[1*]

[1] Laboratory of Cellular and Molecular Biology, Department of Clinical Pathology, National Institute of Gastroenterology, "Saverio de Bellis" Research Hospital, Castellana Grotte 70013, BA, Italy; maria.refolo@irccsdebellis.it; caterina.messa@irccsdebellis.it; rosalba.dalessandra@irccsdebellis.it; [2] Medical Oncology Unit, National Institute of Gastroenterology, "Saverio de Bellis" Research Hospital, Castellana Grotte 70013; claudio.lotesoriere@irccsdebellis.it; ivan.lolli@irccsdebellis.it;

[*] Correspondence:
caterina.messa@irccsdebellis.it;
rosalba.dalessandro@irccsdebellis.it
Tel.: +39 0804994178;
Laboratory of Cellular and Molecular Biology, Department of Clinical Pathology, National Institute of Gastroenterology, "Saverio de Bellis" Research Hospital, Castellana Grotte 70013, BA, Italy;

Supplementary Material

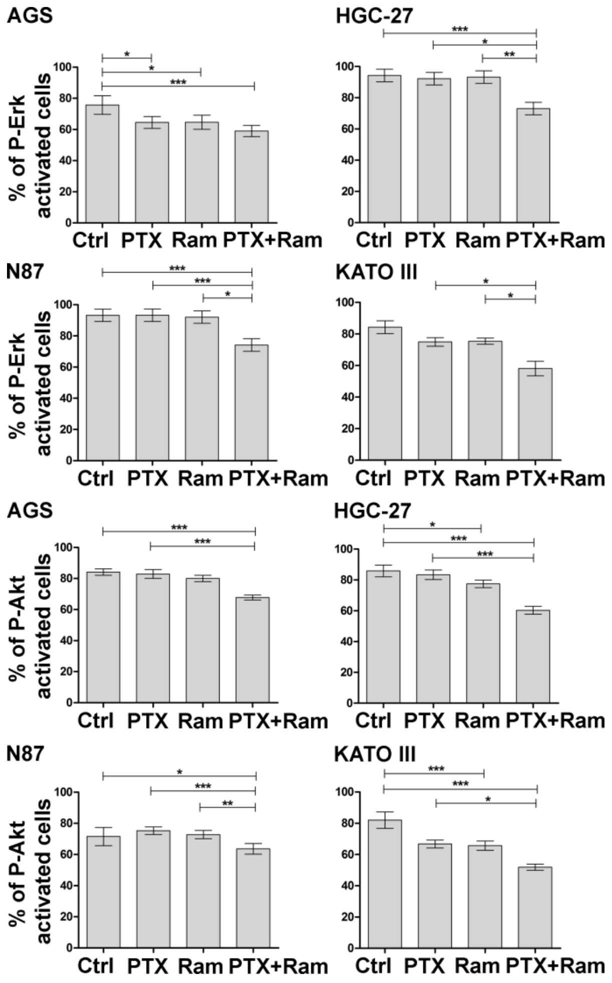

Fig. S1 Ramucirumab and/or Paclitaxel treatment affect MAPK and PI3K/Akt signaling in gastric cancer cell lines. The Muse Activation Kits were used to evaluate the ERK and Akt phosphorylation relative to the total protein expression after 15 min and 24 h respectively in AGS, HGC-27, NCI-N87 [N87] and KATO III cells. Data relative to three independent experiments, expressed as mean ± SD, were plotted in the graphs. *$p<0.05$; **$p<0.001$; ***$p<0.0001$
